# A global view of porcine transcriptome in three tissues from a full-sib pair with extreme phenotypes in growth and fat deposition by paired-end RNA sequencing

**DOI:** 10.1186/1471-2164-12-448

**Published:** 2011-09-10

**Authors:** Congying Chen, Huashui Ai, Jun Ren, Wanbo Li, Pinghua Li, Ruimin Qiao, Jing Ouyang, Ming Yang, Junwu Ma, Lusheng Huang

**Affiliations:** 1 Key Laboratory for Animal Biotechnology of Jiangxi Province and the Ministry of Agriculture of China, Jiangxi Agricultural University, 330045, Nanchang, China

**Keywords:** novel transcript unit, alternative splicing, gene boundary, miRNA, differentially expressed gene, complex trait, pig

## Abstract

**Background:**

Elucidation of the pig transcriptome is essential for interpreting functional elements of the genome and understanding the genetic architecture of complex traits such as fat deposition, metabolism and growth.

**Results:**

Here we used massive parallel high-throughput RNA sequencing to generate a high-resolution map of the porcine mRNA and miRNA transcriptome in liver, longissimus dorsi and abdominal fat from two full-sib F_2 _hybrid pigs with segregated phenotypes on growth, blood physiological and biochemical parameters, and fat deposition. We obtained 8,508,418-10,219,332 uniquely mapped reads that covered 78.0% of the current annotated transcripts and identified 48,045-122,931 novel transcript fragments, which constituted 17,085-29,499 novel transcriptional active regions in six tested samples. We found that about 18.8% of the annotated genes showed alternative splicing patterns, and alternative 3' splicing is the most common type of alternative splicing events in pigs. Cross-tissue comparison revealed that many transcriptional events are tissue-differential and related to important biological functions in their corresponding tissues. We also detected a total of 164 potential novel miRNAs, most of which were tissue-specifically identified. Integrated analysis of genome-wide association study and differential gene expression revealed interesting candidate genes for complex traits, such as *IGF2, CYP1A1, CKM *and *CES1 *for heart weight, hemoglobin, pork pH value and serum cholesterol, respectively.

**Conclusions:**

This study provides a global view of the complexity of the pig transcriptome, and gives an extensive new knowledge about alternative splicing, gene boundaries and miRNAs in pigs. Integrated analysis of genome wide association study and differential gene expression allows us to find important candidate genes for porcine complex traits.

## Background

The pig has been providing with large scale products for human consumption. It also services as an important animal model for human diseases including obesity, diabetes, cardiovascular disease and endocrinology because of the similarity in physiology, organ development and disease progression [1]. However, until now, only several causative mutations have been isolated for porcine complex traits, e.g. *IGF*2 for muscle growth and fat deposit [2], *PRKAG*3 for glycogen content in skeletal muscle [3] and *NR6A*1 for number of vertebrae [4]. Elucidating the complexity of the pig transcriptome is not only essential for interpreting the functional elements of the genome, but also benefits the understanding of human related complex traits such as fat deposition, metabolism and growth. For example, UTR lengths are correlated with gene function, localization and requirements for regulation [5]; alternative splicing plays a major part in biological complexity in humans [6]; and non-coding RNAs (ncRNA) are crucial for multiple biological processes [7,8].

The global transcriptome profilings in abdominal fat, induced fat cells, muscle, liver and pituitary gland have been investigated in pigs by long SAGE analysis, full-length enriched cDNA library and microarray [9-11]. However, the complexity of the porcine transcriptome is not yet fully elucidated. The massively parallel sequencing using next generation sequencer (RNA-seq) provides a huge potential to revolutionize the field of pig transcriptome, owning to its abilities to discover extensive alternative splicing and identify large-scale novel transcripts at single-nucleotide resolution [12-15]. Moreover, the paired-end sequencing strategy of RNA-seq further improves sequencing efficiency and extends short read lengths for better understanding transcriptome [16]. RNA-seq has opened a new horizon for our understanding of global gene expression and has demonstrated the complexity of mammalian transcriptome vastly underestimated before.

MiRNAs are a class of small RNAs that regulate gene expression by decreasing the target mRNA levels or inhibiting translation of protein encoding transcripts. Global miRNA abundance has been measured in skeletal muscle by microarray to evaluate the roles of miRNAs in pig development and meat production [17-19]. MiRBase 15.0 database has collected 189 porcine mature miRNAs. RNA-seq also provides valuable insights into miRNA transcriptome, especially into those miRNAs insufficiently detected by microarray. Until now, porcine miRNA transcriptome has been investigated by next generation sequencer in intestine [20], pre- and postnatal piglet [21], developing brain [22] and skeletal muscle [23].

We herein performed a global transcriptome analysis on three types of tissues related to metabolism, meat production and fat deposition: liver, longissimus dorsi muscle (LD) and abdominal fat (AF) harvested from a full-sib F_2 _female pair with extreme phenotypes by RNA-seq. The results allowed us to investigate large-scale alternative splicing events, identify extensive number of novel transcript units, determine gene boundaries at single nucleotide resolution and comprehensively survey porcine microRNAs in the tested tissues. To our knowledge, this study presented the first systematical investigation on the complexity of porcine transcriptome with nucleotide resolution. Moreover, this study identified many important candidate genes related to growth, meat quality, blood physiological and biochemical parameters by the integrated analysis of genome-wide association study and differential gene expression.

## Results

### Sequencing and mapping of the porcine transcriptome

We sequenced cDNA libraries from 3 tissues of the full-sib female pair using High-seq 2000 at BGI-Shenzhen, China. The data set was analyzed according to the BGI bioinformatics protocols for RNA-seq. The sequence reads have been submitted to the NCBI Gene Expression Omnibus under accession no. GSE26572. In total, we acquired 38,808,956-40,133,362 paired-end reads of 90 bp. The total read length was 21.3 gigabases (Gb), representing about 8-fold of the porcine genome size. We technically replicated the RNA-Seq experiments in the 6 samples with 0.90 <*R^2 ^*< 0.93 (Additional file 1). Furthermore, the expression patterns of 16 randomly selected transcripts between two individuals were validated by qRT-PCR with a relative coefficient of *R^2 ^*= 0.8 (Additional file 1). The technical replicates and qRT-PCR confirmed the high reproducibility of RNA-seq in this study.

We aligned all short reads onto the whole reference genome (Sscrofa9.2). Tolerances were set to allow at most two mismatches for 90 bp reads in each alignment. About 61.4-65.6% of reads were mapped to the pig reference genome, of which 60.2-74.9% fell in annotated exons; 24.1-38.3% located in introns; 0.04-0.06% overlapped with exons, and the remaining 0.8-1.4% were assigned to intergenic regions (Sscrofa9.2). Total 53.1-60.8% of reads had a uniquely genomic location, and 47.9-63.1% of reads corresponded to reference genes with 21.3-25.5% of uniquely matched reads. Unmapped or multi-position matched reads (39.2-46.9%) were excluded from further analyses (Table 1).

**Table 1:** Summary of the numbers of reads, identified genes, novel transcribed units, alternative splicing genes and extended genes

	2268 AF	2268 liver	2268 LD	2270 AF	2270 liver	2270 LD
total reads	40,000,000	38,808,956	39,020,950	40,000,000	40,133,362	39,164,798
total unmapped reads	14,293,709	13,771,813	14,076,474	15,445,291	14,285,367	13,457,962
reads perfectly matched to the reference genome	16,867,579	17,398,322	12,737,195	15,882,824	17,575,361	13,920,553
reads with < = 2 bp mismatch to the reference genome	8,838,712	7,638,821	12,207,281	8,671,885	8,272,634	11,786,283
reads uniquely matched to the reference genome	22,908,741	22,998,328	23,301,854	21,235,855	23,695,323	23,823,564
reads matched to the reference genome with multi-positions	2,797,550	2,038,815	1,642,622	3,318,854	2,152,672	1,883,272
annotated genes identified in this study	19,062	17,382	15,612	18,695	17,447	15,716
reads uniquely matched to annotated genes	8,508,418	9,465,873	8,615,406	9,075,466	10,219,332	9,530,691
reads matched to annotated genes with multi-positions	10,648,618	15,009,898	13,906,877	10,839,003	15,072,157	13,441,905
novel transcript units	122,931	71,394	48,045	112,052	72,401	69,677
clustered transcriptionally active regions	29,499	22,871	17,085	25,842	21,840	22,099
alternative splicing genes	2882	1,851	1,859	2,832	2,116	1,657
5' extended genes	815	610	587	793	697	593
3' extended genes	1,201	1,375	1,075	1,360	1,224	1,064
Genes extended both 5' and 3' ends	672	551	432	636	590	411

### Identification of an extensive number of novel transcript units

**Figure 1:**
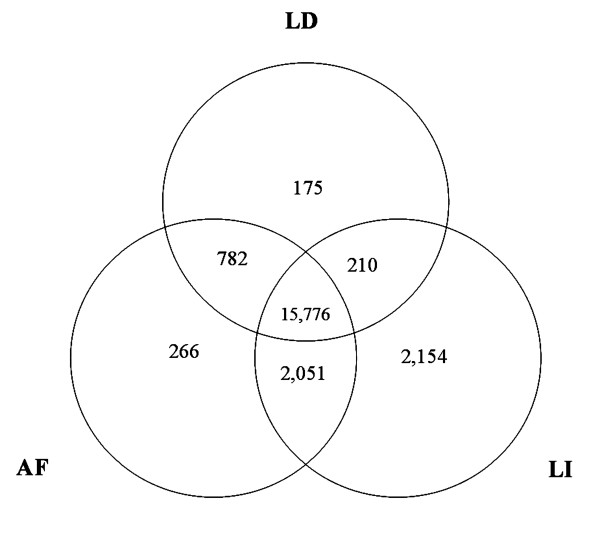
**Comparison of the identified genes among liver, LD and AF against the UCSC database**. The number in overlapped regions is the annotated genes that were expressed in all three tissues or each two tissues.

The uniquely mapped reads (8,508,418-10,219,332) covered 78.0% (21,414/27,444) of the annotated transcripts in UCSC pig genome database [24] by at least one sequence read. A total of 15,776 transcripts were expressed in all three tissues, and 266, 175 and 2,154 transcripts were discovered exclusively in AF, LD and liver, respectively (Figure 1). We quantified the gene expression level by counting the number of reads per kilobase per million mapped reads (RPKM). About 74.6-84.3% of the annotated transcripts showed expression with > 0.5 RPKM. The obtained transcription fragments of more than 99.9% of the detected transcripts were > 150 bp in length (Additional file 2). The percentage of a gene covered by reads was defined as gene coverage. Extensive read mapping revealed about 40.3-50.9% of the detected transcripts with more than 50.0% in gene coverage.

We detected an extensive number of novel transcript units by the procedures described in Methods and Zhang et al. (2010) [25]. In total, we obtained 122,931, 71,394, 48,045, 112,052, 72,401 and 69,677 novel transcript units, respectively, in the six tested samples with a mean length of 285 bp and a size range from 150 to 39,638 bp (Table 1). Many novel transcript units (27.7-85.1%) have > 1 exons and the largest one is 39,638 bp in length containing 136 exons (Additional file 3). The novel transcript units were often identified in clusters, indicating that closely spaced transcript units are likely to merge into longer transcripts with increasing sequencing depth. By scanning the genomic location of each novel transcribed unit, we clustered adjacent fragments (± 3 kb) into one transcriptionally active region. In this way, we identified 17,085-29,499 novel transcriptionally active regions in the six tested samples (Table 1). A proportion (3.6-16.9%) of the regions was comprised of single exon.

To investigate whether the identified novel transcript units were non-coding RNAs, we aligned the sequences of the novel transcript units to non-coding RNA precursor sequences in Rfam database [26]. We found that 0.12-0.17% of novel transcript units were non-coding RNA precursors. Of these, 47.0% were in average tRNA precursors, 26.2% were miRNA precursors and 15.6% were snoRNA precursors.

### Alternative splicing events in pig transcriptome

**Figure 2:**
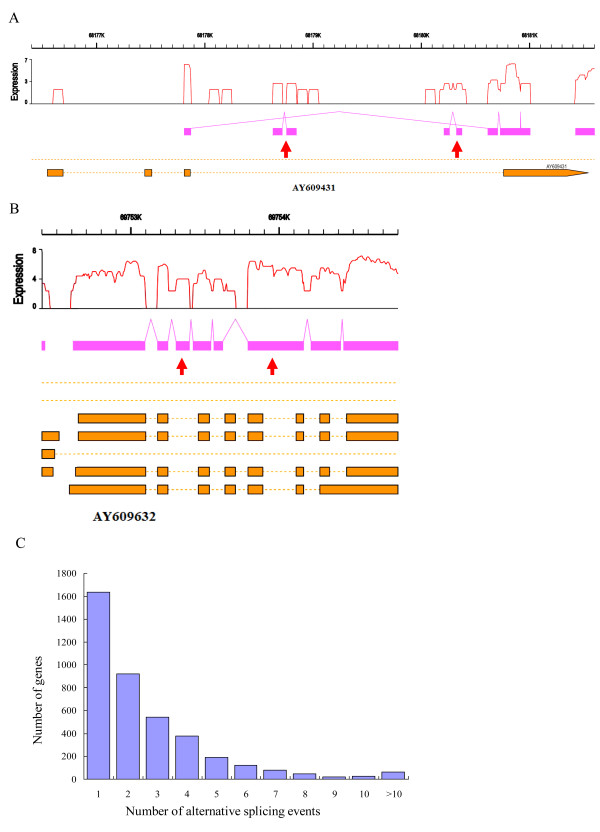
**An example of read distributions of genes in reference genome**. The schematic diagram depicts the read distributions of AY609431 (A) and AY609632 (B) on pig chromosomes 3 and 2. The red curve shows the expression level (log2 of RPKM) and the pink bar denotes the genomic regions covered by reads in RNA-seq. The yellow bar shows the transcript structures of genes in UCSC. The pink lines highlight the linkage between exons which were supported by at least two distinct junction reads. The red arrows indicate the alternative 5' splicing in panel A and the retained intron in panel B. The plot (C) shows the distribution of the number of alternative splicing events occurred in each gene and including all four types of alternative splicing models in individual 2268.

To investigate alternative splicing, we identified the sequence reads that were mapped to the regions of computationally determined theoretical splicing junctions. Four known types of alternative splicing models including alternative 3' splicing site, alternative 5' splicing site, exon skipping and intron retention were considered in this study. The distribution of alternative splicing events is shown in Additional file 4. The alternative splicing events from 3 tissues of one individual were pooled and the redundancy was removed to get a final set of alternative splicing events. We found that up to 4,038 genes accounting for 18.9% of the known genes had undergone alternative splicing in these tissues, displaying 10,746 alternative events in individual 2268. Similarly, the number of alternative splicing genes and events in individual 2270 were 4,024 (18.8% of the known genes) and 10,854, respectively (Table 1 and Additional file 5). Figure 2A and 2B show an example of intron retention and alternative 5' splicing site. We found that about 59.0% of the alternative spliced genes underwent multiple alternative splicing events (Figure 2C), illustrating the complexity of porcine transcriptome. Alternative 3' splicing site is the most common type of alternative splicing events accounting for 40.8% of all alternative splicing events in pigs, while intron retention and exon-skipping only constituted 7.5% and 15.6% in individual 2268, or 7.4% and 15.9% in individual 2270. The average size of retained introns is 536 bp, ranging from 54 bp to 9691 bp.

### Extension of annotated gene boundaries

The extensions of 5' and 3' boundaries were determined by comparison of the gene models obtained by RNA-seq with the existing gene annotations. In the six tested samples, a total of 587-815 genes were extended at the 5' end, of which more than 65.3% had an extension of at least 50 bp in length. In comparison, 1,064-1,375 genes were extended at their 3' end, of which more than 71.0% were extended by at least 50 bp. Furthermore, 411-672 genes were extended at both ends (Table 1 and Additional file 6). In individual 2268, total 1,399 annotated genes were extended at the 5' end, of which 10.9% (152) were observed in all three tissues; and 2,505 annotated genes were extended at the 3' end, of which 16.0% (401) had the extended 3' boundary in all three tissues. Similarly, in individual 2270, the percentage of the extended 5' or 3' genes shared in all three tissues was 10.3% (147) and 17.0% (260), respectively.

### Comprehensive survey of porcine microRNA by deep sequencing

**Figure 3:**
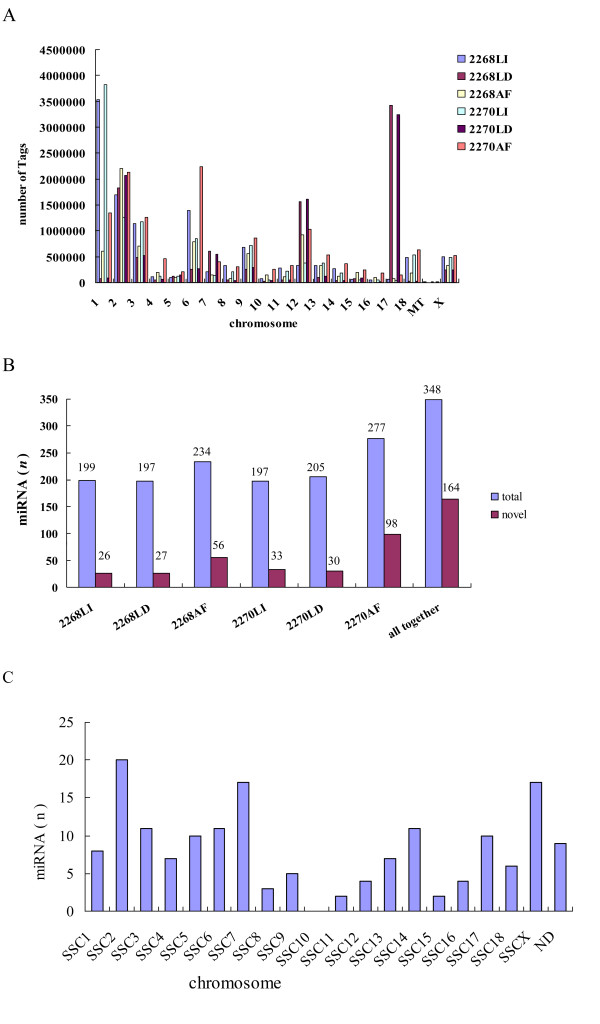
**Discovery of miRNAs**. (A) The graph shows the abundance of small RNA tags mapped to each chromosome. (B) The number of the identified mature miRNAs and putative novel miRNAs. (C) The number distribution of the putative novel miRNAs mapped to each chromosome. Abbreviation: ND, not determined.

To get a comprehensive view of miRNA transcriptome in pigs, we carried out deep sequencing of small RNA (18-30 nt) using the tested samples mentioned above. The obtained miRNA sequence reads have been submitted to the NCBI Gene Expression Omnibus under accession no. GSE26572. We obtained a total of 8,951,703-13,479,372 raw reads. After removing low quality reads and corrupted adapter sequences, 7,282,608-11,208,822 clean reads were retained for further analyses. The majority of small RNA was 20-23 nt for all libraries (> 84.0%), with 22 nt small RNA being the most abundant (Additional file 7), which is in agreement with the common nucleotide length of miRNAs. We mapped 69.2-83.6% of clean reads to the reference genome (Sscrofa9.2). Chromosomes 1, 2, 3, 6, 12 and 17 harbored clean reads of > 1,000,000 tags (Figure 3A). After further removal of tRNA, rRNA, scRNA, snRNA, snoRNA, exon RNA, intron RNA and repeat regions, a total of 4,484,788-7,226,415 miRNA sequences were obtained (Additional file 8).

**Figure 4:**
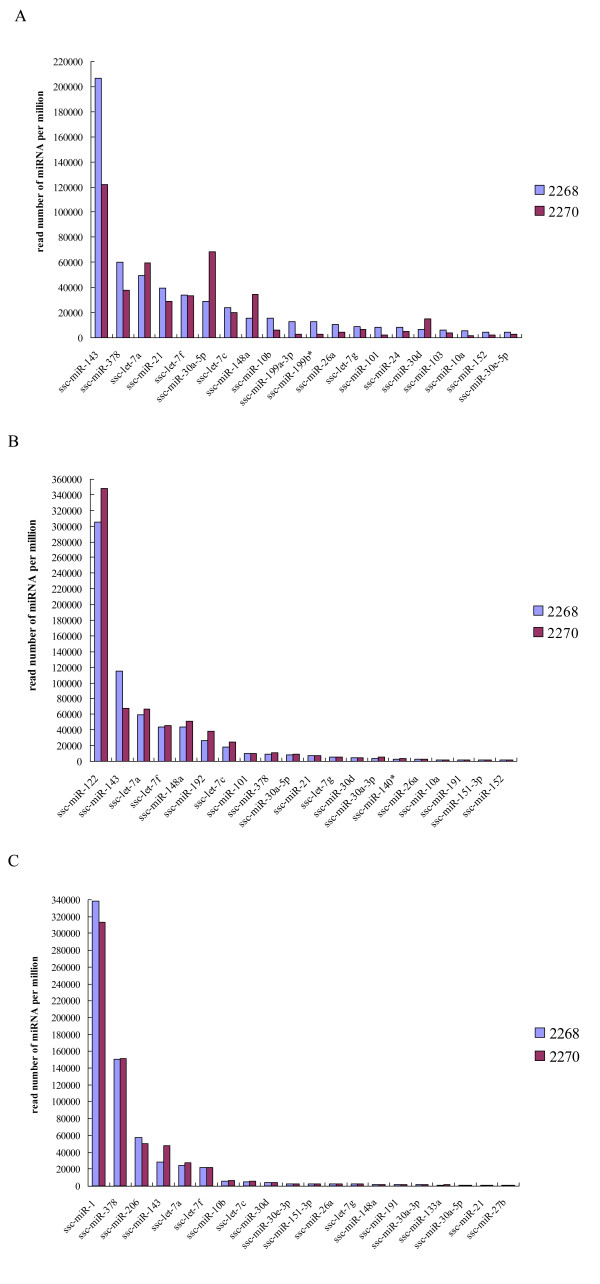
**The mature miRNAs ranking top 20 in normalized expression levels in each sample**. AF (A), liver (B) and LD muscle (C).

Aligning miRNA sequences to the porcine mature miRNAs in miRBase 15.0 database [27] revealed 86.8 (164/189)-94.7 (179/189)% of mature miRNAs in each sample. We pooled all identified mature miRNAs and found that only five mature miRNAs in miRBase 15.0 database were not detected in this study (Figure 3B). The expression levels of the identified miRNAs displayed a very large range, as reflected by the number of sequence reads, which varied from single counts for rare miRNAs to several hundred thousand reads for the most abundant miRNAs (Additional file 9). As many as 87.3-98.4% of the clean reads belong to the miRNAs ranked top 20 in expression levels in each sample (Figure 4).

To identify potential novel miRNA, we further analyzed the small RNA tags that could not match known miRNAs and were mapped to intergenic or intronic regions of the reference genome. The characteristic of hairpin structure of miRNA precursor was used to predict novel miRNA by exploring the secondary structure. In total, 26 to 98 potential novel miRNAs each supported by at least five sequence reads, were identified in the 6 tested samples (Figure 3B). The potential novel miRNAs of 6 samples were pooled and the redundancy was removed to get a final set of 164 unique porcine putative novel miRNAs. The vast majorities of these miRNAs were expressed at low levels. Nevertheless, 6 miRNAs showed relatively high expression levels representing by more than 1,000 sequence reads, and 22 by more than 100 sequence reads (Additional file 10).

To determine the genomic locations of these potential novel miRNAs, their precursor sequences were blasted against the porcine reference genome sequence (Sscrofa9.2). As shown in Figure 3C, 94.5% of the novel miRNAs were assigned to the reference genome. Most of the precursors were located on chromosome 2 (n = 20) followed by chromosomes × and 7 (n = 17), and none of the novel miRNAs were mapped to chromosome 10.

### Cross-tissue comparison of differential transcription events

Deep RNA sequencing in three types of tissues allows us to investigate the tissue-differentially transcriptional events. Of the 21,414 identified transcripts, 266, 175 and 2,154 transcripts were discovered exclusively in AF, LD and liver, respectively (Figure 1). Interestingly, as reported in humans [14], the majority of alternative splicing events showed clear tissue specificity, demonstrating the importance of alternative splicing in tissue specific programs of gene expression and its major roles in functional complexity. Functional annotations of alternative splicing genes showed that they play important roles in their corresponding tissues. The porcine *RYR1 *gene (M91451) which had the largest number of LD-specific alternative splicing events has been associated with malignant hyperthermia and has significant effects on pig meat quality and carcass leanness [28]. The *ALB *(AK232454) gene having the most number of liver-specific alternative splicing events plays important roles in transportation of fatty acids [29]. *SLA-1 *(AK231553) implicating in immune and type I diabetes [30] had the most number of alternative splicing events in AF that has been known as an important immune organ. Other tissue-specifically alternative splicing genes also show major roles in their corresponding tissues. For instance, *MyHC-2A *(AB025260), *PPAP2C *(FJ436381) and *APOH *(AK232456) had tissue-specific alternative splicing in LD, AF and liver, respectively, corresponding to their roles in normal muscle development and function [31], converting phosphatidic acid to diacylglycerol [32], transportation of fatty acids [29], and lipoprotein metabolism [33] (Additional file 11).

The expression abundance of many miRNAs also showed apparently tissue-differential patterns. The mature miRNAs with high abundance in each tissue are conserved in mammals and likely related to important biological functions. MiR-122 is the most abundant miRNA in human liver and also had the most abundance of expressed read counts in porcine liver (Figure 4). MiR-122 plays a crucial role in cholesterol, fatty acid and lipid metabolism [34-36]. The miR-1 and miR-206 are key mediators in proper skeletal and cardiac muscle development and function, myogenesis during embryonic development and muscle cell differentiation [37]. The two miRNAs, respectively, had the most and third abundance of expression level in LD in this study. For those potential novel miRNAs identified in this study, 85 of 164 putative novel miRNAs were specifically identified in AF, 23 and 24 in liver and LD, respectively (Table 2). Their biological functions need further investigation.

**Table 2:** The identified putative novel miRNAs

	Liver-specific	AF-specific	LD-specific	in total
2268-specific	7	17	6	30
2270-specific	15	52	14	81
Both in 2268 and 2270	1	16	4	21
novel miRNAs identified in more than one tissues				28
novel miRNAs identified in all samples				4
total number of novel miRNA	23	85	24	164

### Differentially expressed transcripts and miRNAs between two individuals

**Figure 5:**
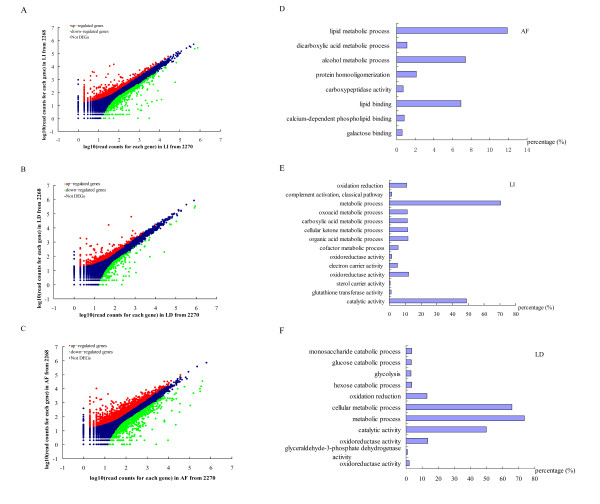
**Analyses of differentially expressed genes between individuals 2268 and 2270**. The graphs of (A) to (C) show differentially expressed genes. The up-regulated genes and the down-regulated genes are respectively shown in red and green. Genes with similar expression levels are indicated in blue. The graphs of (D) to (F) indicate the GO analyses of differentially expressed genes; the bar plot represents the percentage of gene counts within each GO category. All functions or processes listed have enrichment *P *values < 0.05.

Overall, there were clear linear relationships in the gene expression levels (0.84 <*R^2 ^*< 0.88) between two individuals in all three tissues. The number of unique reads mapped to different genes ranged from 1 to 894,235. The differentially expressed genes were selected based on the expression profiles and the following criteria: (1) if the fold change in gene expression levels between 2268 and 2270 was more than or equal to two fold (log_2_-fold change ≥ 1 or ≤ -1) and (2) if the false discovery rate value was less than 0.001. With this, we identified 2,796, 1,551 and 835 differentially expressed genes in AF, liver and LD, respectively. Of these, the expressions of 1,997, 825 and 505 genes were up-regulated in 2268 with respect to individual 2270 (Figure 5A-5C and Additional file 12).

To further investigate the biological relationships of differentially expressed genes with phenotypes, we performed the gene ontology (GO) analysis by querying each differentially expressed gene into the records of the GO database [38]. The results of GO functional annotations are presented in Figure 5D-5F. The main functional groups of differentially expressed genes in AF are related to lipid metabolic process, alcohol metabolic process, lipid binding and protein homooligomerization. The functions of differentially expressed genes in liver are enriched in metabolic process, catalytic activity and oxidoreductase activity. And the differentially expressed genes in LD are mainly associated with metabolic process, cellular metabolic process, catalytic activity and oxidoreductase activity.

Differentially expressed miRNAs between two individuals were identified by comparing the normalized expression data of the mature miRNAs. In total, 10 differentially expressed miRNAs (fold-change (log2) ≥ 1 or fold-change (log2) ≤ -1; *P*-value < 0.01) were identified in liver, 20 and 63 in LD and AF, respectively. Most of the differentially expressed miRNAs had relatively low expression levels (Additional file 13). Interestingly, some differentially expressed miRNAs are involved in the pathway relevant to development and diabetes. For instance, miR-214 differentially expressed in LD enables precisely specific the muscle cell types by sharpening cellular responses to Hedgehog in Zebrafish [39]; a liver-differentially expressed miRNA of miR-10b is predicted to regulate genes in pathways relevant to type 2 diabetes [40].

### Investigation of candidate genes for related phenotypes by integrated analysis of genome-wide association study (GWAS) and differential gene expression

**Figure 6:**
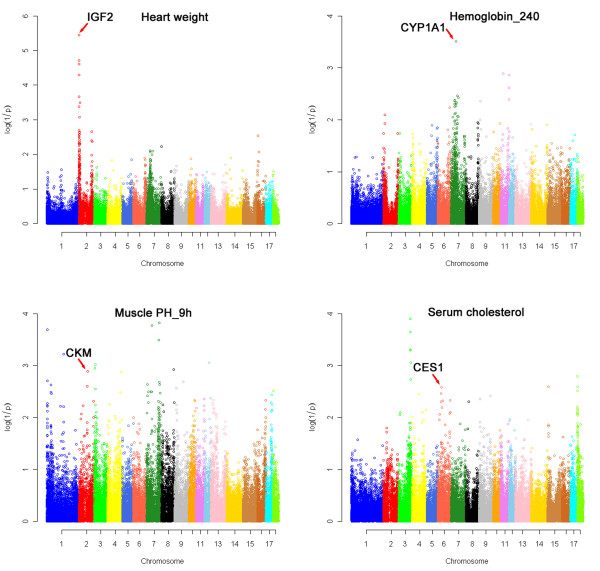
**Identification of candidate genes related to phenotypes by integrated analysis of GWAS and differentially expressed genes**. *IGF2, CYP1A1, CKM *and *CES1 *locate at the QTL region for heart weight, hemoglobin level at day 240, pork pH value at 9 h after slaughter and serum total cholesterol, respectively. The *X*-axis indicates the chromosome regions of SNPs, and the *Y*-axis shows the log10 radios of corrected *P*-value.

We selected total 500 most differentially expressed genes including 200 from liver and AF, respectively, 100 from LD for further functional annotations (if false discover rate of 1 × 10^-50 ^was set as the threshold for selecting the transcripts for further analyses, only 100 of 835 differentially expressed genes could be chosen from LD). Only 359 genes could be mapped to the pig reference genome (Sscrofa9.2) and had functional annotations in mammals. Of the 359 genes, 142 have the description of phenotypes in knocked-out mice [41]. A genome-wide association study using the pig 60K SNP chip has been performed in the current White Duroc × Erhualian F_2 _resource population (unpublished data). Seven, 11 and 4 differentially expressed genes in liver, AF and LD are located within 2.5 Mb around the SNPs that were most significantly associated with phenotypes in the GWAS and the associated phenotypes were also observed in the corresponding gene-deficient mice (Additional file 12). Here, we present the identification results of interesting candidate genes *IGF2, CYP1A1, CKM *and *CES1 *for the related phenotypes by integrating the analysis of GWAS and differential gene expression in Figure 6.

### Confirmation of *IGF2 *as a causative gene for heart weight

*IGF2 *is the causative gene underlying the QTL for muscle growth, fat deposition and heart weight on SSC2p [2]. A nucleotide substitution in intron 3 of *IGF2 *abrogates interaction with ZBED6, resulting in threefold increase of *IGF2 *messenger RNA expression [42]. In the current F_2 _population, a significant QTL for heart weight at day 240 was located at chr2: 1.3 Mb with a corrected *P*-value of 3.6 × 10^-6 ^in GWAS (Figure 6). This region overlapped with the *IGF2 *locus. In the RNA-seq analysis, individual 2268 with the heavier heart weight (307.5 g vs. 225.0 g) showed a 2.3 fold increase in *IGF2 *expression level compared with individual 2270. The result was perfectly consistent with the previous result [2], and is another example demonstrating the causality of this gene in the QTL.

### *CYP1A1 *as a strong candidate gene for hemoglobin

Two founder breeds of the F_2 _resource population are White Duroc and Chinese Erhualian, which are divergent in hemoglobin concentration [43]. Blood parameters of hemoglobin and hematocrit at day 240 were measured in the F_2 _population. We identified a significant QTL for both hemoglobin and hematocrit content at day 240, which is located at chr7: 66.9 Mb in GWAS (*P *= 4.9 × 10^-5^). A differentially expressed gene of *CYP1A1 *in liver by RNA-seq is located within this QTL region. The *CYP1A1 *knocked-out mice had the increased hemoglobin content [44]. A quantitative trait transcript (QTT) analysis in this F_2 _population also revealed that the *CYP1A1 *expression level was associated with hemoglobin at day 240 (*P *= 2.2 × 10^-3^, unpublished data). These results strongly support *CYP1A1 *as a candidate for the QTL effect on hemoglobin content.

### *CKM *as a candidate gene for pork pH value

Creatine kinase, muscle (*CKM*) is a cytoplasmic enzyme involved in energy homeostasis. *CKM*-deficient mice show abnormal muscle physiology and an increased skeletal muscle glycogen level [45]. Excess glycogen content in pig skeletal muscle is associated with low meat pH value, leading to bad meat quality [3]. A significant QTL for meat pH value at 45 min, 3 h and 9 h after slaughter was detected around the region of chr2: 80.0 Mb with a *P*-value of 1.3 × 10^-3 ^in the F_2 _cross. *CKM *gene locates at the chr2: 79.1 Mb and was differentially expressed in this full-sib pair with different meat pH values. This indicates that *CKM *is an important candidate gene for meat pH value in pigs.

### *CES1 *as an important candidate gene for serum cholesterol and triglyceride level

The liver is an important organ for lipid metabolism. Gene expression in liver influences the circulating cholesterol level. *CES1 *that participates in fatty acyl and cholesterol ester metabolism [46] was one of the differentially expressed genes in liver. Our previous QTT study in liver showed that the expression level of *CES1 *is significantly associated with serum total cholesterol (*P *= 0.02) and triglyceride level (*P *= 7.1 × 10^-3^) in the F_2 _population (Unpublished data). A significant QTL for both low density lipoprotein cholesterol and total cholesterol was identified at chr6: 18.5 Mb containing the *CES1 *gene in GWAS. The concordant results of QTT, GWAS and differential expression suggest that *CES1 *is strong candidate gene for QTL affecting serum total cholesterol on chromosome 6. The supporting evidence was also from the *CES1 *knocked-out mice that showed decreased circulating cholesterol level [47].

## Discussion

In this study, we presented the systematical transcriptome profiling of pigs on three tissues related to metabolism, meat production and fat deposition using high throughput RNA-seq technology. This efficient deep sequencing not only allows us to analyze novel transcribed regions and miRNAs, but also improves gene annotations at single nucleotide resolution. Integrated analysis of genome-wide association study and differential gene expression between two individuals revealed important candidate genes for related phenotypes.

A total of 38,808,956-40,133,362 reads were obtained from RNA-seq in six samples with 53.1-60.8% of reads uniquely mapped to the reference genome. This data set provided comprehensive starting resources for improving the gene annotations across the porcine genome. High *R^2 ^*values between technical replicates showed high reproducibility of RNA-seq in pigs. It should be mentioned that there are still 34.4-38.6% of reads that cannot be matched to the reference genome. This could be caused by low sequence coverage of the reference genome, reference errors, sequencing errors and defined mapping criterions. A proportion (4.2-8.3%) of mapped reads with multiple positions was discarded, attributing to known duplicated genes or chromosome segments. A majority of the annotated transcripts (78.0%) at UCSC database [24] was covered by sequence reads, showing the sensitivity of RNA-seq in transcript discovery even for lowly expressed genes [48]. Furthermore, we identified large number of novel transcript units which improved the gene annotations of the porcine genome and transcriptome.

Alternative splicing is an important model of gene expression regulation and has not been generally accessible for microarray or SAGE methods in pigs. Some genes showed all four types of alternative splicing models (such as *CSN1S1*), revealing the complexity of alternative splicing in pigs. We found that alternative 3' splicing is the most common type of alternative splicing events in pigs. This is in contrast to the report in human and yeast where exon-skipping is the most prevalent mechanism [13,14], and it is also different from rice in which intron retention is the primary alternative splicing type [25]. The percentage of alternative 5' or 3' splicing in total alternative splicing events in this study were higher than that reported in Lim et al. (2009) [49] where 8.0% of alternative 5' splicing and 3% of alternative 3' splicing were observed. We discovered that more than 18.0% of the detected genes were alternatively spliced. This number is much lower than the reported 86.0% in human [14] and 33.0% in rice [25]. Three types of alternative splicing models including alternative first exon, alternative last exon and mutually exclusive exon were excluded from analyses because of currently unperfected algorithms. More alternative spliced genes would be discovered if these alternative splicing models were considered.

As an important regulator of gene expression, miRNA regulates the gene expression through decreasing the target mRNA levels or repressing the translation [50,51]. In this study, the high identification rate illustrates that the six small RNA libraries from the tested tissues almost encompass the entire repertoire of known miRNAs. Consistent with the results in Li et al. (2010) [21], porcine mature miRNAs had a broad range of expression levels. The highly expressed miRNAs are known to have important regulatory functions in corresponding tissues. The miR-1/206 showed high abundance of expression levels in muscle (Figure 3C). Interestingly, muscular hypertrophy in Texel sheep has been shown to be caused by a mutation that creates an illegitimate binding site for miR-1/206 in the 3' UTR of the myostatin gene, leading to efficient translational inhibition of the myostatin gene and an increase in muscularity [8]. The miR-122 had the most abundant expression level in liver. It has the diversity of its roles in liver, e.g. metabolism, hepatocarcinogenesis [34]. Just as in humans [52], most of the potential novel miRNAs discovered in this study are expressed at low levels (Additional file 10), explaining why they were not discovered in previous efforts and showing the advantage of RNA-seq in transcriptome analysis. Furthermore, we found that most of the novel miRNAs were tissue-specifically identified (Table 2). We cautioned that some could be artifact due to the low expression level, the insufficient depth of sequencing and limited tested tissues.

Mapping genetic factors that underlie quantitative traits in farm animals has been a challenging task [53]. The recent wave of genome-wide association studies in human showed that a majority of SNPs associated with disease traits locate in regulated regions [54]. Integration of gene expression with genotype and phenotype data to elucidate the network of molecular interactions that underlie complex traits can facilitate the identification of variants that contribute to phenotypes [54]. In this study, high-throughput sequencing allowed us to digitally discover an extensive number of differentially expressed genes in the full-sib pair with different phenotypes. Gene ontology analysis indicated that the differentially expressed genes had enrichment on functions related to metabolic process, catalytic activity and lipid binding. The results suggest that these differentially expressed genes are likely related to the phenotypes of growth, metabolism or fat deposition. By integrating GWAS, differentially expressed genes and altered phenotypes in knocked-out mice, we found that many differentially expressed genes are the important candidate genes related to the phenotypes of serum cholesterol, growth traits, hemoglobin at day 240, fatty acid level and muscle pH value (Additional file 14). For example, activating transcription factor 4 (ATF4) gene differentially expressed in muscle and located in the QTL region for body weight at day 210 is related to decreased body weight in ATF4 knocked-out mice (Table S12) [55]; fatty acid binding protein 4 (FABP4) located in the QTL region for fat deposition on SSC4 and related to increased white adipose tissue amount in FABP4 knocked-out mice was differentially expressed in abdominal fat in this study (Table S12) [56]. The findings provide important clues for further dissecting of the responsible genes and variants.

## Conclusions

This study provides a global view of the complexity of the pig mRNA and miRNA transcriptome, gives an extensive new knowledge about alternative splicing, novel transcript units, gene boundaries and novel miRNAs in pigs. The cross-tissue comparison identified lots of tissue-differential transcription events. Integrated analysis of GWAS and differential gene expression allowed us to detect important candidate genes related to growth, meat quality, serum lipids and fatness. The findings significantly enhance the current genome annotation of pigs and improve our understanding of complex traits.

## Methods

### Animals and sample collection

Two F_2 _full-sib females from a White Duroc × Erhualian resource population were used in this study. All animals were housed in a consistent and standard environmental condition. The room temperatures were uncontrolled with natural lighting. Animals were floor fed three times a day. The phenotypes of growth (body weight at birth, day 46, 210 and 240), pork pH value at 45 min, 3 h, 9 h, 15 h and 24 h after slaughter, blood physiological and biochemical parameters including serum cholesterol, hemoglobin and blood cell, carcass traits and meat fatty acid level were measured as described previously [57,58]. The full-sib pair had different phenotypes, such as extremely phenotypic distribution in growth and fatness (Additional file 15). Liver, LD and AF from both individuals were harvested for RNA isolation within 30 mins after slaughter at the age of 240 day. All animal procedures were conducted according to the guidelines for the care and use of experimental animals established by the Ministry of Agriculture of China.

### RNA isolation and quality assessment

Total RNA was isolated with TRIzol (invitrogen) according to the manufacture's instructions. DNA was removed from RNA extracts with RNase-free DNase I (New England Biolabs) for 30 min at 37°C. The quality of total RNA was assessed by the 2100 Bioanalyzer (Agilent) and agarose gel electrophoresis.

### cDNA library construction and sequencing

Poly (A) mRNA was isolated from the total RNA samples with oligo (dT) magnetic beads (invitrogen). Purified mRNA was first fragmented by the RNA fragmentation kit (Ambion). The first-strand cDNA synthesis was performed using random hexamer primers and reverse transcriptase (invitrogen), and the second-strand cDNA was synthesized using RNase H (invitrogen) and DNA polymerase I (New England Biolabs). The cDNA libraries were prepared using the Illumina Genomic DNA Sample Prep kit (Illumina) following the manufacturer's protocol, and then loaded onto flow cell channels of the Illumina High-seq 2000 platform for paired-end 90 bp × 2 sequencing. The average insert size for the paired-end libraries was 200 bp (from 180 to 220 bp). Total six paired-end cDNA libraries were constructed each for six tested samples (tissues of liver, LD and AF from 2268 and 2270). One technical replicate was performed for each sample.

### Small RNA library preparation and sequencing

Small RNA libraries were constructed according to the Illumina alternative v1.5 protocol for small RNA sequencing. Briefly, small RNA sized at 18-30 nt was purified from total RNA through polyacrylamide gel electrophoresis, and 3' and 5' Illumina RNA adapters were ligated to the small RNA molecules by T4 RNA ligase (New England Biolabs). The ligated small RNA was subsequently transcribed into cDNA and then amplified for 15 cycles with PCR using primers corresponding to the ends of the adapters. After purified with gel, the amplified cDNA constructs were sequenced according to the Illumina GA platform sequencing protocols.

### Mapping reads to the porcine reference genome and annotated transcripts

The porcine reference genome sequence and annotated transcript set were downloaded from the UCSC (Sscrofa9.2) [24]. After removing reads of low quality (more than half of the base's qualities were less than 5), reads containing Ns > 5 and reads containing adapters, clean reads were aligned to the porcine reference genome using SOAP2 [59] allowing up to two mismatches in 90-bp reads. For the reads that were unalignable to the reference sequences, SOAP iteratively trim several base pairs at the 3'-end and redo the alignment, until a match was detected or the remaining sequence was too short for specific alignment. A similar strategy was used to align reads to the porcine annotated transcript set. The different insert size between paired reads (1 bp -10 kb for mapping to genome, and < 1 kb for mapping to genes) was set to align the exon-exon junction reads.

### Identification of novel transcript units

All reads that matched to the reference genome with multi-positions were excluded for further analysis. The intergenic regions were defined within the 200-bp down stream of one gene to the 200-bp up stream of the next adjacent gene using the porcine mRNA data (UCSC). A contiguous expression region with each base supported by at least two reads was considered as a transcriptionally active region (TAR). The TARs that were joined by at least one set of paired-end reads were connected into a transcript unit. Those transcript units that were not overlapped with an annotated gene model and located in intergenic regions with a continuous mapping length ≥ 150 bp and average coverage ≥ 2 were considered as the putative novel transcript units. To determine whether the novel transcript units were non-coding RNAs, we blasted the sequences of the novel transcript units with RNA families in Rfam database (version 10.0) by rfam _ scan.pl (1.0.2) with default threshold [26,60].

### Identification of alternative splicing

To identify potential splicing sites, all putative junction sites which give information about boundaries and combinations of different exons in a transcript were determined by TopHat [61]. All reads that did not match to the genome were aligned onto the splice junctions to identify the junction reads. A junction site was required to be supported by at least two unambiguously mapped reads with non-repetitive match position within the splice and having a minimum of five bases on both sides of the junction.

As described by Wang et al. (2008) [14] and Zhang et al. (2010) [25], and according to the structures of exons, the alternative splicing events were classified into seven different types of alternative splicing models including alternative 3' splice site, alternative 5' splice site, exon skipping, intron retention, alternative first exon, alternative last exon and mutually exclusive exon. The details of these alternative splicing models were described in Zhang et al. (2010) [25]. Because of the unperfected algorithms for alternative first exon, alternative last exon and mutually exclusive exon, only the remaining four alternative splicing models listed above were analyzed and presented in this study.

### Determination of gene boundary

The gene structure was optimized according to the distribution of the reads, paired-end sequences and the annotation of reference genes. After alignment of reads to the reference genome, the genomic regions with continuous reads and uniquely mapped reads ≥ 2 formed transcription active regions. We connected the different transcription active regions to form a potential gene model using the paired-end data. The extensions of 5' and 3' boundaries were determined by comparison of the potential gene model with the existing gene annotation.

### Differentially expressed genes analysis

Numbers of reads per kilobase of exon region in a gene per million mapped reads were used as the value of normalized gene expression levels [12]. Differentially expressed genes and their corresponding *P*-values were determined with methods described by Audic and Claverie (1997) [62]. The significance threshold of *P*-value in multiple tests was set by false discovery rate (FDR). The fold changes (log2Ratio) were also estimated according to the normalized gene expression level in each sample. We use "FDR ≤ 0.001 and the absolute value of log2Ratio ≥ 1" as the threshold to judge the significance of gene expression difference.

### Gene ontology annotation

The differentially expressed genes were classified for the categories of molecular function, cellular component and biological process using gene ontology (GO) annotation. Hypergeometric test was applied to map all differentially expressed genes to terms in GO database [38] and search significantly enriched GO terms in differentially expressed genes comparing to the genome background. The calculated *P*-values were corrected through bonferroni correction, taking corrected-*P *value ≤ 0.05 as a threshold of significance.

### Real-time quantitative RT-PCR (qRT-PCR)

As the current gold standard for quantification of mRNA, to validate the repeatability and reproducibility of gene expression data obtained by RNA sequencing in pigs, we performed qRT-PCR on 16 randomly selected genes including 7 differentially expressed genes with the total RNA used in RNA-seq. The first-strand cDNA was synthesized with superscript II reverse transcriptase (Invitrogen). Gene-specific primers were designed according to the gene sequence using primer premier 5.0 (Additional file 16). The *GAPDH *gene was used as a control in the experiments. qRT-PCR was carried out in triplicate with Power SYBR Green Mastermix (Applied Biosyetems Inc.) on an Applied Biosystems Step One Plus system using the following program: 95°C for 5 min; 35 cycles of 95°C for 15 sec, 60°C for 15 sec, and 72°C for 40 sec; 72°C for 6 min.

### Discovery and annotation of miRNA

Raw tag sequences were produced by the Illumina Genome Analyzer II at BGI-Shenzhen, China and the data set was analyzed according to the BGI bioinformatics protocols for small RNA. Briefly, the low quality tags and adaptor contaminants formed by adaptor ligation from the 35 nt tags were first filtered from the data set. We then summarized the length distribution of the clean tags and retained only short trimmed reads of sizes from 18 to 30 nt.

The distributions of small RNA tags on the reference genome were mapped by SOAP2. Those tags matched with rRNA, scRNA, snoRNA, snRNA and tRNA in Genbank and Rfam database or aligned to exonic and repetitive regions (release 9.0) [26] were excluded from advanced analyses. To determine mature miRNAs, the unique small RNA tags were aligned with the known miRNAs of pigs in miRBase15.0 database [27] with a maximum of two mismatches. For those unannotated small RNA tags that could be mapped to intergenic or intronic regions, Mireap was used to predict potential novel miRNAs by exploring the secondary structure, the dicer cleavage site and the minimum free energy of the precursors [25].

The expressions of miRNAs were normalized to get the expression level of transcripts per million. The differentially expressed miRNAs were determined by calculating the fold-change (log 2 ratios) and *P*-value from the normalized expression. Fold-change (log2) ≥ 1 or fold-change (log2) ≤ -1 and *P*-value < 0.01 were used as the thresholds to judge the significance of differentially expressed miRNA.

### Integrated analysis of GWAS and differential gene expression

A panel of F_2 _animals (n = 933) was successfully genotyped using Porcine 60K SNP chips (Illumina) and an internally developed SNP set. The genomic position of each SNP (Sscrofa9.2) was determined by SOAP2. The quality control of genotypes was performed with GenABEL procedure in *R*. The associations of the genome-wide SNP genotype data with phenotypic traits were analyzed with PLINK, and the significant *P*-values were adjusted by bonferroni correction.

The genomic locations of differentially expressed genes were determined by BLAT [63]. Because of the extensive linkage disequilibrium in F_2 _crosses, the differentially expressed genes located within 2.5 Mb around the most significant SNPs were selected for further functional annotation. The database in Mouse Genome Informatics [41] was used to search phenotypes linked with differentially expressed genes in knocked-out mice.

## List of Abbreviations

AF: abdominal fat; *ALB*: Albumin; *APOH*: apolipoprotein H; *CES1*: carboxylesterase 1; *CKM*: creatine kinase, muscle; *CSN1S1*: casein alpha-S1; *CYP1A1*: cytochrome P450, family 1, subfamily A, polypeptide 1; *GAPDH*: glyceraldehyde-3-phosphate dehydrogenase; *IGF2*: Insulin-like growth factor 2; LD: longissimus dorsi muscle; *MyHC-2A*: myosin heavy chain 2a; *PPAP2C*: phosphatidic acid phosphatase type 2C; *RYR1*: ryanodine receptor 1; *SLA-1*: MHC class I antigen 1; ZBED6: zinc finger, BED-type containing 6.

## Authors' contributions

LSH, conceived and designed the experiments, revised the manuscript; CYC, performed the experiments, analyzed the data, wrote and revised the manuscript; HAS, analyzed the data; JR, provided comments and suggestions for the manuscript; WBL, revised the manuscript; PHL, RMQ, JO and MY, RNA extraction; JWM: genotyped the F_2 _individuals with 60K SNP chip. All authors read and approved the final manuscript.

## Supplementary Material

Additional file 1 **Figure S1 and S2**. Repeatability of technical replicates in RNA-seq by comparing the gene expression levels. Figure S1, Scatterplots comparing the gene expression levels (Log10 (read count)) based on technical replicates of LD and LI from both individuals. Figure S2, Comparison of the expression ratios of randomly selected genes between two individuals obtained by RNA sequencing and qRT-PCR, respectively. The *X*-axis and *Y*-axis show the log2 radios of gene expression levels of the 2 tested animals determined by qRT-PCR and High-seq 2000, respectively. Click here for file

Additional file 2 **Table S1**. Gene expression levels and coverage of transcripts identified in each sample. Click here for file

Additional file 3 **Table S2**. Novel transcript units identified in each sample. Click here for file

Additional file 4 **Table S3**. Alternative splicing events identified in each sample. Click here for file

Additional file 5 **Table S4**. Summary of the number of each type of alternative splicing events in different tissues and individuals. Click here for file

Additional file 6 **Table S5**. The detailed description of the extension of gene boundary in each sample. Click here for file

Additional file 7 **Figure S3**. The distribution of the nucleotide length of small RNAs. (A) 2268 AF; (B) 2268 LI; (C) 2268 LD; (D) 2270 AF; (E) 2270 LI; (F) 2270 LD. Click here for file

Additional file 8 **Table S6**. Distribution of the number of small RNA tags among different categories. Click here for file

Additional file 9 **Table S7**. The expression levels of the mature miRNAs in each tissue. Click here for file

Additional file 10 **Table S8**. Identification of novel miRNAs and its expression levels in each sample. Click here for file

Additional file 11 **Table S9**. Functional annotations of some tissue-specifically alternative splicing genes. Click here for file

Additional file 12 **Table S10**. Differentially expressed genes between two individuals in each tissue. Click here for file

Additional file 13 **Table S11**. Differentially expressed miRNAs identified in each tissue. Click here for file

Additional file 14 **Table S12**. Associations of differentially expressed genes with phenotypes. Click here for file

Additional file 15 **Table S13**. The phenotypic values of individuals 2268 and 2270. Click here for file

Additional file 16 **Table S14**. Primers for qRT-PCR. Click here for file
