# Supplementary material for: A global view of porcine transcriptome in three tissues from a full-sib pair with extreme phenotypes in growth and fat deposition by paired-end RNA sequencing
Source: BMC Genomics. 2011 Sep 10;12:448. doi: 10.1186/1471-2164-12-448 (PMC3188532; doi:10.1186/1471-2164-12-448)

**Figure legends**

**Figure S1.** **Scatterplots comparing the gene expression levels**

The repeatability of RNA-seq was evaluated by comparing the gene expression levels (Log10 (read count)) based on technical replicates of LD and LI from both individuals.

**Figure S2. Comparison of the expression ratios of randomly selected genes using RNA sequencing and qRT-PCR.**

The *X*-axis and *Y*-axis shows the log2 radios of gene expression levels of the 2 tested animals determined by qRT-PCR and High-seq 2000, respectively.


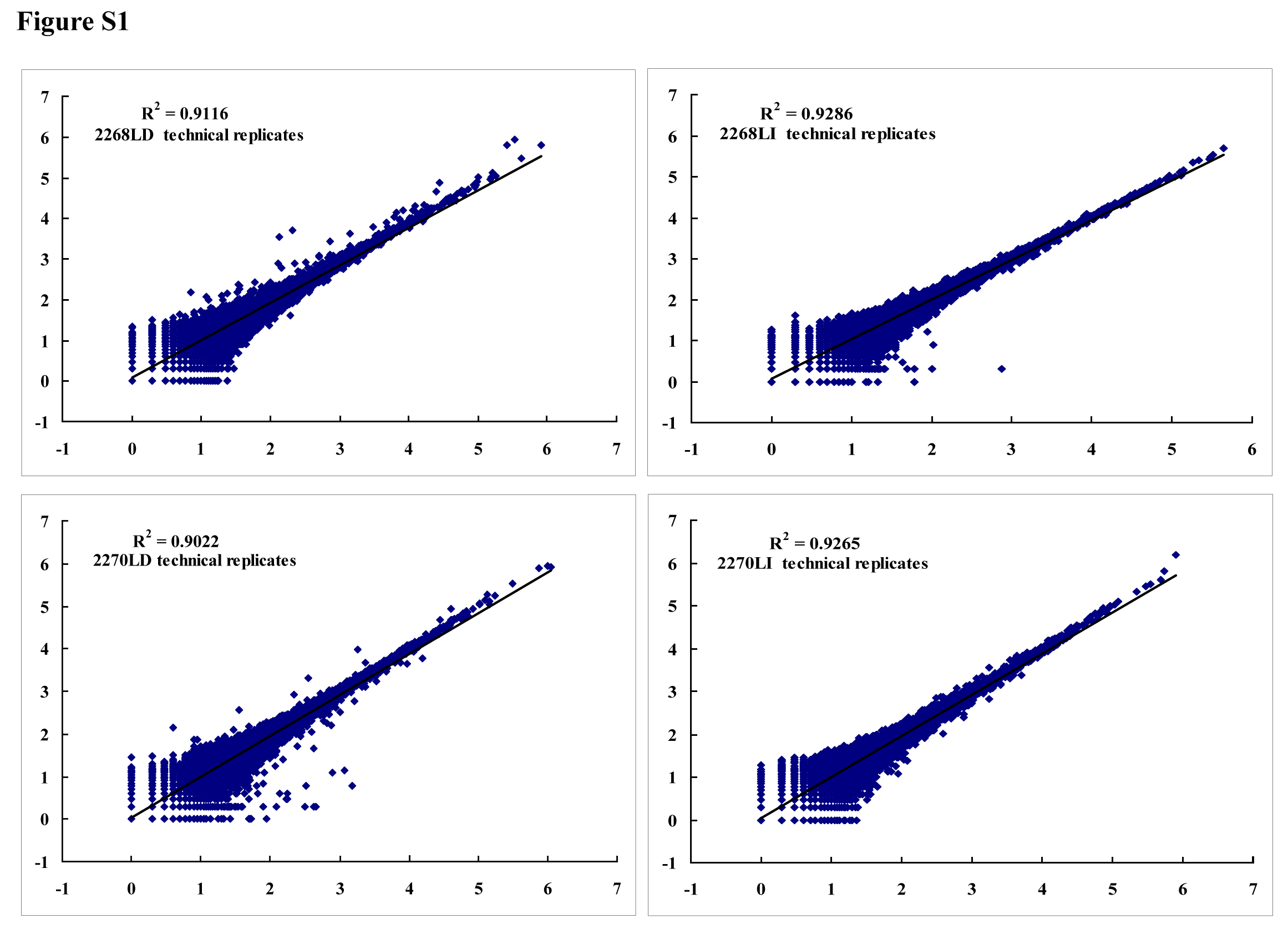


**Figure S2**


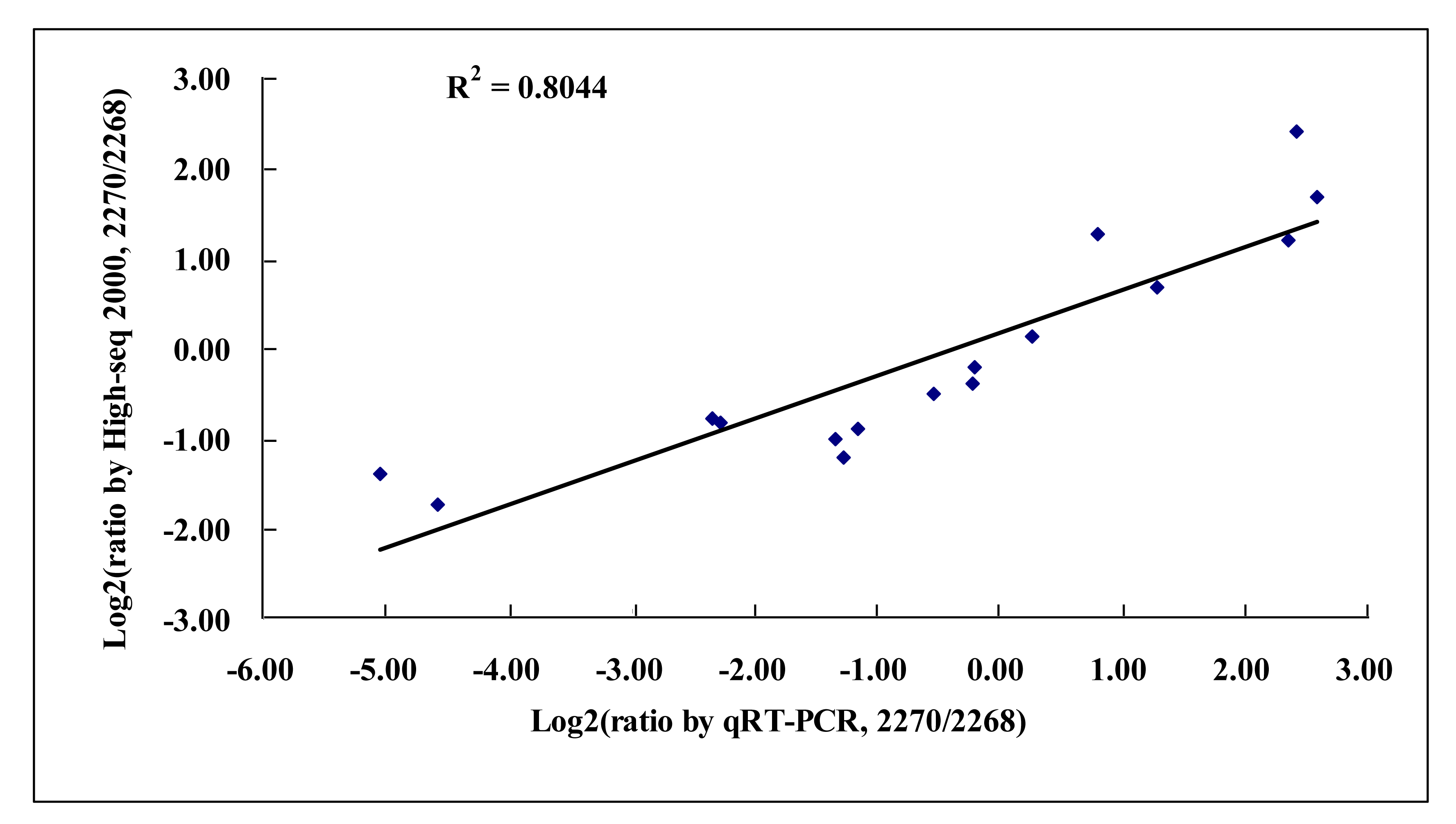

Supplement: Additional file 1 — Figure S1 and S2. Repeatability of technical replicates in RNA-seq by comparing the gene expression levels. Figure S1, Scatterplots comparing the gene expression levels (Log10 (read count)) based on technical replicates of LD and LI from both individuals. Figure S2, Comparison of the expression ratios of randomly selected genes between two individuals obtained by RNA sequencing and qRT-PCR, respectively. The X-axis and Y-axis show the log2 radios of gene expression levels of the 2 tested animals determined by qRT-PCR and High-seq 2000, respectively. [file 1471-2164-12-448-S1.DOC]
