# Supplementary material for: A global view of porcine transcriptome in three tissues from a full-sib pair with extreme phenotypes in growth and fat deposition by paired-end RNA sequencing
Source: BMC Genomics. 2011 Sep 10;12:448. doi: 10.1186/1471-2164-12-448 (PMC3188532; doi:10.1186/1471-2164-12-448)
Supplement: Additional file 5 — Table S4. Summary of the number of each type of alternative splicing events in different tissues and individuals. [file 1471-2164-12-448-S5.DOC]

**Table S4.** The number of each type of alternative splicing events in different tissues and individuals

|  | **2268** | | | | | | | |  | **2270** | | | | | | | |
| --- | --- | --- | --- | --- | --- | --- | --- | --- | --- | --- | --- | --- | --- | --- | --- | --- | --- |
|  | **AF** | | **LI** | | **LD** | | **individual^1^** | |  | **AF** | | **LI** | | **LD** | | **individual** | |
|  | genes | events | genes | events | genes | events | genes | events |  | genes | events | genes | events | genes | events | genes | events |
| A3SS | 1,582 | 2,303 | 1,024 | 1,642 | 1,091 | 1,728 | 2,502 | 4,385 |  | 1,539 | 2,290 | 1,261 | 1,963 | 951 | 1,462 | 2,487 | 4,434 |
| A5SS | 1427 | 1,982 | 967 | 1,453 | 948 | 1,449 | 2,266 | 3,859 |  | 1,333 | 1,882 | 1,134 | 1,766 | 866 | 1,289 | 2,305 | 3,895 |
| ES | 721 | 908 | 413 | 574 | 473 | 609 | 1,202 | 1,672 |  | 742 | 996 | 471 | 649 | 394 | 499 | 1,206 | 1,723 |
| IR | 566 | 794 | 36 | 46 | 25 | 32 | 590 | 830 |  | 544 | 763 | 48 | 58 | 25 | 31 | 565 | 802 |
| Total^2^ | 2882 | 5,987 | 1,851 | 3,715 | 1,859 | 3,818 | 4,038 | 10,746 |  | 2,832 | 5,931 | 2,116 | 4,436 | 1,657 | 3,281 | 4,024 | 10,854 |

Note: A3SS: alternative 3’ splice site; A5SS: alternative 5’ splice site; ES: exon skipping; IR: intron retention; AF: abdominal fat; LD: longissimus dorsi muscle; LI: Liver; 1: combined: combined analysis of alternative splicing events in all three tissues. The genes which had the same alternative splicing event in more than on organ were counted only once in individual. 2: some genes had all four types of alternative splicing events in one individual.
