# Supplementary material for: A global view of porcine transcriptome in three tissues from a full-sib pair with extreme phenotypes in growth and fat deposition by paired-end RNA sequencing
Source: BMC Genomics. 2011 Sep 10;12:448. doi: 10.1186/1471-2164-12-448 (PMC3188532; doi:10.1186/1471-2164-12-448)
Supplement: Additional file 7 — Figure S3. The distribution of the nucleotide length of small RNAs. (A) 2268 AF; (B) 2268 LI; (C) 2268 LD; (D) 2270 AF; (E) 2270 LI; (F) 2270 LD. [file 1471-2164-12-448-S7.DOC]

**Additional figure 3.** **The distribution of the nucleotide length of small RNAs**.

(A) 2268 AF; (B) 2268LI; (C) 2268LD; (D) 2270AF; (E) 2270LI; (F) 2270LD

**
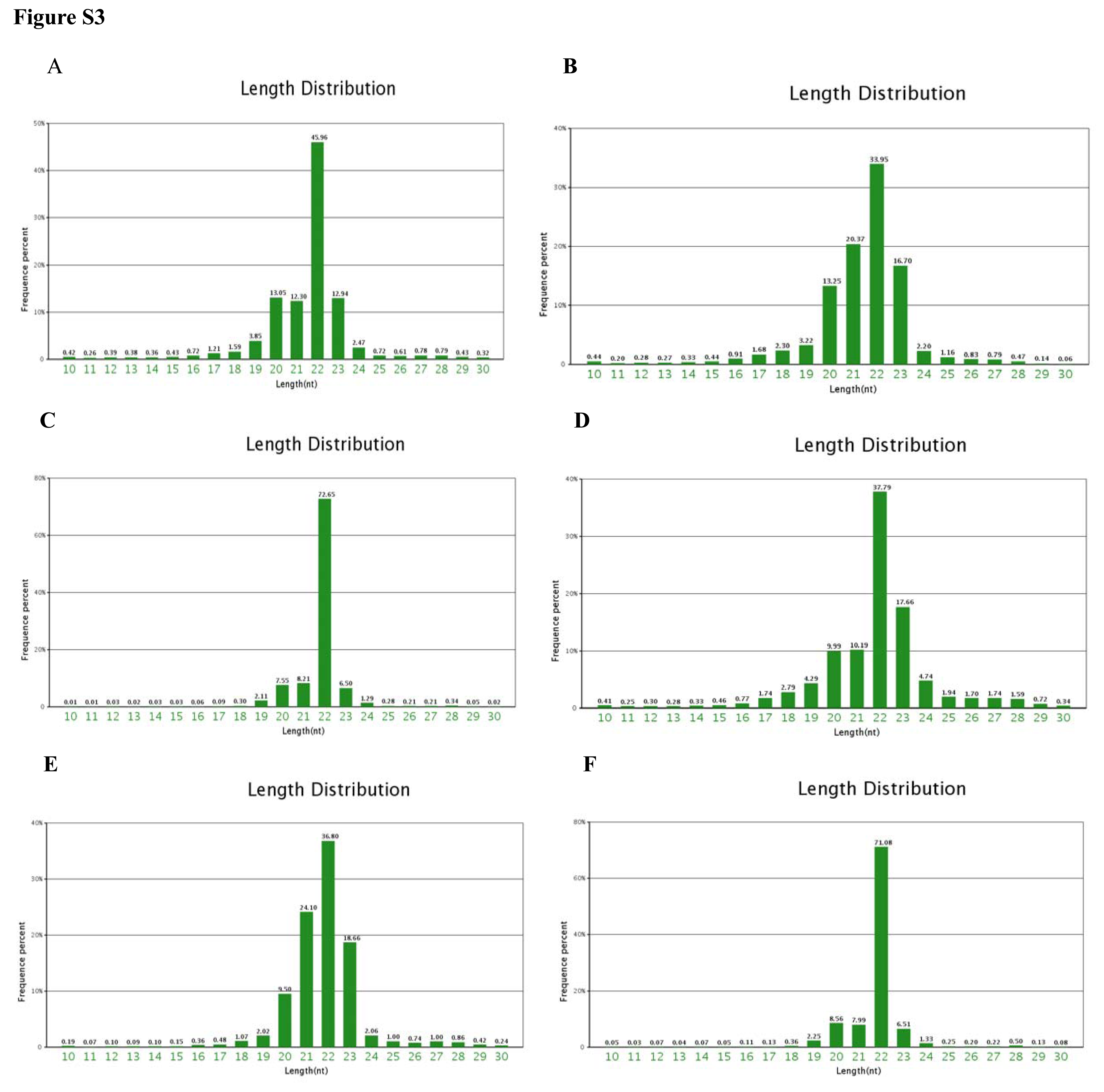
**
