# Supplementary material for: A global view of porcine transcriptome in three tissues from a full-sib pair with extreme phenotypes in growth and fat deposition by paired-end RNA sequencing
Source: BMC Genomics. 2011 Sep 10;12:448. doi: 10.1186/1471-2164-12-448 (PMC3188532; doi:10.1186/1471-2164-12-448)
Supplement: Additional file 11 — Table S9. Functional annotations of some tissue-specifically alternative splicing genes. [file 1471-2164-12-448-S11.DOC]

**Table S9.** The functional annotations of the parts of tissue-specifically alternative splicing genes

| **Accession No. in UCSC** | **Gene** | **Number of AS events** | **Specificity in tissue** | **Function** | **Reference** |
| --- | --- | --- | --- | --- | --- |
| AB025260 | myosin heavy chain 2a (*MyHC-2A*) | 16 | Longissimus dorsi muscle | Myosin heavy chain 2a is necessary for normal muscle development and function. | Tajsharghi et al. 2010 |
| M91451 | Sus scrofa ryanodine receptor (*RYR1*) | 40 | Longissimus dorsi muscle | associated with malignant hyperthermia and had significant effects on pig meat quality and on carcass leanness | Fujii et al. 1991; Stinckens et al. 2009 |
| AK232454 | Albumin (*ALB*) | 34 | Liver | Albumin functions primarily as a carrier protein for steroids, fatty acids, and thyroid hormones | Baker 2002 |
| AK232456 | apolipoprotein H (*APOH*) | 10 | Liver | implicated in a variety of physiologic pathways including lipoprotein metabolism | Kamboh and Ferrell 1991; Yasuda et al. 2000 |
| AK231553 | MHC class I antigen 1 (*SLA-1*) | 33 | Abdominal fat | Involved in immune and type I diabetes | Barrett et al*.* 2009 |
| FJ436381 | phosphatidic acid phosphatase type 2C (*PPAP2C*) | 8 | Abdominal fat | converting phosphatidic acid to diacylglycerol, and function in de novo synthesis of glycerolipids as well as in receptor-activated signal transduction | Long et al. 2008 |

Baker. 2002. Albumin, steroid hormones and the origin of vertebrates, *J Endocrinol* **175:** 121-127.

Barrett JC, Clayton DG, Concannon P, Akolkar B, Cooper JD, Erlich HA, Julier C, Morahan G, Nerup J, Nierras C, et al. 2009. Genome-wide association study and meta-analysis find that over 40 loci affect risk of type 1 diabetes. *Nat Genet* **41**: 703-707.

Fujii J, Otsu K, Zorzato F, de Leon S, Khanna VK, Weiler JE, O'Brien PJ, MacLennan DH. 1991. Identification of a mutation in porcine ryanodine receptor associated with malignant hyperthermia. *Science* **253**: 448-451.

Kamboh MI, Ferrell RE. 1991. Apolipoprotein H polymorphism and its role in lipid metabolism. *Adv Lipid Res* **1**: 9–18.

Long JS, Pyne NJ, Pyne S. 2008. Lipid phosphate phosphatases form homo- and hetero-oligomers: catalytic competency, subcellular distribution and function. *Biochem J* **411:** 371-377.

Stinckens A, Luytena T, Van den Maagdenberg K, Janssens S, De Smet S, Georges M, Buys N. 2009. Interactions between genes involved in growth and muscularity in pigs: IGF-2, myostatin, ryanodine receptor 1, and melanocortin-4 receptor. *Domest Anim Endocrinol* **37**: 227-235.

Tajsharghi H, Hilton-Jones D, Raheem O, Saukkonen AM, Oldfors A, Udd B. 2010. Human disease caused by loss of fast IIa myosin heavy chain due to recessive MYH2 mutations. *Brain* **133:**1451-459.

Yasuda S, Tsutsumi A, Chiba H, Yanai H, Miyoshi Y, Takeuchi R, Horita T, Atsumi T, Ichikawa K, Matsuura E, Koike T. 2000. beta (2)-glycoprotein I deficiency: prevalence, genetic background and effects on plasma lipoprotein metabolism and hemostasis. *Atherosclerosis* **152**: 337-346.
