# Supplementary material for: A global view of porcine transcriptome in three tissues from a full-sib pair with extreme phenotypes in growth and fat deposition by paired-end RNA sequencing
Source: BMC Genomics. 2011 Sep 10;12:448. doi: 10.1186/1471-2164-12-448 (PMC3188532; doi:10.1186/1471-2164-12-448)
Supplement: Additional file 15 — Table S13. The phenotypic values of individuals 2268 and 2270. [file 1471-2164-12-448-S15.DOC]

**Table S13.** The phenotypic values of the individual 2268 and 2270

| **ID** | **Mother** | **Father** | **Gender** | **Weight of Leaf Fat (g)** | **Weight of Abdominal Fat (g)** | **Body weight at Day 210 (Kg)** | **Meat PH _9h^1^** | **Heart weight (g)** | **TC (g/L)^2^** | **Hemoglobin at day 240 (g/L)** |
| --- | --- | --- | --- | --- | --- | --- | --- | --- | --- | --- |
| 2268 | 42 | 3 | female | 2842.50 | 1480.00 | 97.60 | 6.45 | 307.50 | 0.07 | 164 |
| 2270 | 42 | 3 | female | 977.50 | 980.00 | 68.70 | 6.26 | 225.00 | 0.21 | 151 |

Note: 1. meat PH_9h: meat PH value at 9 h after freshly slaughtered;

2. TC: total cholesterol.
